# Discrete Convolution-Based Energy Spectrum Configuring Method for the Analysis of the Intrinsic Radiation of ^176^Lu

**DOI:** 10.3390/s21217040

**Published:** 2021-10-23

**Authors:** Kilyoung Ko, Hyunwoong Choi, Yongsun Yi, Wonku Kim, Junhyeok Kim, Jisung Hwang, Eunbie Ko, Gyuseong Cho

**Affiliations:** 1 Department of Nuclear and Quantum Engineering, Korea Advanced Institute of Science and Technology, Daejeon 34141, Korea; coltom@kaist.ac.kr (K.K.); chw1994@kaist.ac.kr (H.C.); kwk94@kaist.ac.kr (W.K.); covent17@kaist.ac.kr (J.K.); jshwang93@kaist.ac.kr (J.H.); cutsky@kaist.ac.kr (E.K.); 2 Department of Nuclear Engineering, Khalifa University of Science Technology and Research, Abu Dhabi P.O. Box 127788, United Arab Emirates; yongsun.yi@ku.ac.ae

**Keywords:** intrinsic radiation, cascade gamma rays, lutetium-based scintillator, silicon photomultiplier, spectrum convolution, genetic algorithm

## Abstract

There has been considerable interest in inorganic scintillators based on lutetium due to their favorable physical properties. Despite their advantages, lutetium-based scintillators could face issues because of the natural occurring radioisotope of ^176^Lu that is contained in natural lutetium. In order to mitigate its potential shortcomings, previous works have studied to understand the energy spectrum of the intrinsic radiation of ^176^Lu (IRL). However, few studies have focused on the various principal types of photon interactions with matter; in other words, only the full-energy peak according to the photoelectric effect or internal conversion have been considered for understanding the energy spectrum of IRL. Thus, the approach we have used in this study considers other principal types of photon interactions by convoluting each energy spectrum with combinations for generating the spectrum of the intrinsic radiation of ^176^Lu. From the results, we confirm that the method provides good agreement with the experiment. A significant contribution of this study is the provision of a new approach to process energy spectra induced by mutually independent radiation interactions as a single spectrum.

## 1. Introduction

**Figure 1:**
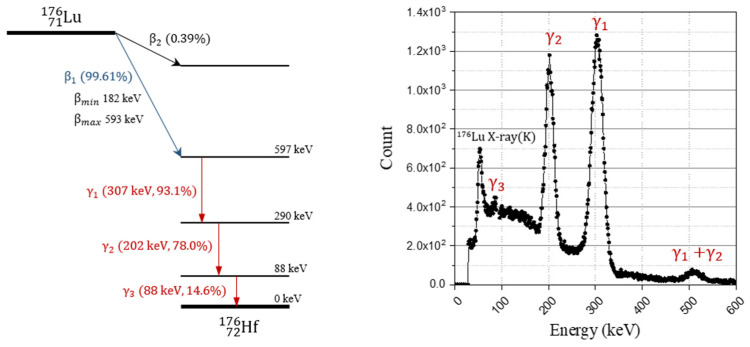
Decay scheme of ^176^Lu and energy spectrum of gamma rays by emitted by decay of ^176^Lu (measured using 2 inch × 2 inch NaI(Tl) scintillator).

There has been considerable interest in inorganic scintillators based on lutetium, such as lutetium oxyorthosilicate (LSO) and lutetium yttrium oxyorthosilicate (LYSO), for use in positron emission tomography (PET) due to their favorable physical properties including high detection efficiency (≈0.86 cm^−1^, linear attenuation coefficient at 511 keV), fast decay time (≈40 ns), and high light yield (≈80% NaI (Tl)) [1,2]. Despite their advantages, lutetium-based scintillators could face issues in single transmission measurement, low sensitivity imaging, and wide energy window-scanning because of the natural occurring radioisotope of ^176^Lu that is contained in natural lutetium [3,4,5]. As shown in Figure 1, ^176^Lu undergoes decay and emits a beta particle with a mean and maximum energy of 182 and 593 keV, and a cascade of gamma rays or internal conversion electrons with energies of 307, 202, and 88 keV [6]. The intrinsic radiation of ^176^Lu (IRL) could create background noise in apparatuses as a uniformly distributed radiation source in scintillation crystals.

Although the effects of IRL on medical imaging have been studied, they have not been a major concern in common in-vivo situations because the activity is negligibly low compared to the radiopharmaceuticals and the nuclear medicine scanners have background rejection capabilities, such as coincidence-timing windows and energy windows [7,8,9]. However, when used in preclinical PET, in-beam PET, the PET/SPECT system, and cell-trafficking studies under the aforementioned special conditions, these effects become problematic. The approach to the considered issue is not straightforward because of the poor energy resolution of the emission tomography apparatus, especially in silicon photomultiplier (SiPM)-based systems, and sometimes the performance of spectroscopy is not guaranteed for a wide energy range [10]. Moreover, ^176^Lu radioactivity of about 240–300 Bq/cm^3^ [11] with a combination of the beta particle and cascade gamma rays affects the entire range of energy for emission tomography, making this approach more difficult.

However, if we have a deep understanding of the background, some characteristics of IRL could actually be beneficial for a number of applications. For instance, the position of the peaks from the cascade gamma rays can be used as an ideal radioactive source for energy calibration without external sources [12,13,14]. The uniformly distributed background radiation can serve as measured transmission data for performing emission data corrections such as attenuation correction and scatter correction, or as initial values for algorithms [13]. The sum energy peak of cascade gamma rays can be used for monitoring detector calibration shift and for self-calibration of detectors in complex detector systems [14].

In order to mitigate the potential shortcomings and encourage the strengths of lutetium-based scintillators in a variety of fields, the present work will address the self-detection characteristics of the IRL in the scope of energy spectroscopy. Previous works have studied the spectrum of IRL self-detection in the scintillation crystal [14,15,16,17,18]. The typical broad-spectrum of IRL was observed and it indicated that simultaneous detection of the β-particle and γ-rays likely contributes to the broadening of the spectrum [14]. Since the relative intensity of the peaks in the spectrum has not been clearly explained, subsequent studies emphasized this and suggested a method based on distinguishing each event combination and interaction probability [14]. More recently, researchers have demonstrated the dependence of the IRL energy spectrum on the geometry of the scintillator through light transport simulation [16]. Apart from these efforts, studies and validation studies have also been conducted to obtain the background spectrum of IRLs by simulating ion sources in simulation packages, such as Geant4 or Geant4-based code. However, simulations of the complete decay and detection processes using simulation packages can be time-consuming and require an experienced user to correctly set the involved simulation parameters to obtain the correct energy spectrum [19,20].

Few studies have focused on other principal types of photon interactions with matter when the IRL spectrum was obtained with a previously presented method known as analytical calculation; in other words, only the full-energy peak according to the photoelectric effect or internal conversion was considered for understanding the energy spectrum of IRL. The full-energy peak is dominant compared to the Compton region but this may not always be a reasonable assumption when the escape probability of the scattered gamma ray increases depending on the structure of the scintillation crystal. Thus, considering various interactions, even analytical calculation, would represent a more detailed approach for understanding while offering generality in relatively small pixelated scintillators, unlike the bulk scintillators used in previous studies [15,16].

The approach we have used in this study considers principal types of photon interactions without ion source simulation. To this end, using empirical parameters, energy spectra reflecting the variation of each gamma ray interaction at a pixelated scintillation crystal were simulated simultaneously with the process that calculates the relative intensity of interactions, as in previous studies (in Section 2.1 to Section 2.2). Each energy spectrum was then convoluted with combinations as base spectra for generating the IRL spectrum (in Section 2.3). Finally, the generated IRL spectrum was compared to the experiment, ion source, and previous method in order to validate the method we used (in Section 3.3). The aim of this work is to suggest the method that shows how to obtain the more detailed IRL background spectrum using only cascade gamma ray interactions without complex understandings of ion source simulation.

## 2. Materials and Methods

### 2.1. Experimental Environment

Experiments were set up to acquire the empirical parameters used for simulation and to measure the IRL spectrum. The apparatus used in the experiment is part of the Brain-PET, which the KAIST research team has been developing. The detector modules, readout, and experiment environment are described next.

The detector module is assembled with a surface mount silicon photomultiplier (SiPM) array PM3325-WB (KETEK GmbH, Munich, Germany) coupled with optical grease (Eljen Technology, Sweetwater, TX, USA) to an LYSO scintillator crystal (Epic crystal, Shanghai, China). SiPM is an analogue device that consists of thousands of Geiger-mode avalanche photo-diode (GAPD) pixels in its area and receives the sum of the signals in a unit pixel as an output signal to measure the amount of incident light [21]. Square-base prism LYSO crystals with all surfaces polished and their sides covered with enhanced specular reflector film (3M Company, Saint Paul, MN, USA) were used in this study. An 8 × 8 SiPM array model was used and the single-pixel package size was 3.315 × 3.315 mm^2^, the active area was 3 × 3 mm^2^, and the fill factor was 80%. The scintillation crystal was 3 × 3 × 20 mm^3^ and one-to-one coupled with the single pixel of SiPM.

**Figure 2:**
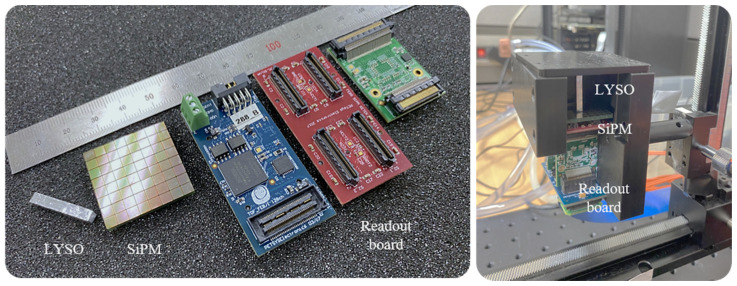
Detector system components (LYSO scintillation crystal (3 × 3 × 20 mm^3^), 8 × 8 array silicon photomultiplier (SiPM), and readout board) (**left**) and experimental setup with light-tight structures (**right**).

A SiPM readout circuit (Front-End Module, PETsys Electronics Inc., Oeiras, Portugal) was used for simultaneous processing of the GAPDs’ charge integration and photo-sensor channel signals. The readout circuit was based on an application-specific integrated circuit (ASIC) in a standard complementary metal-oxide-semiconductor (CMOS). The ASIC digitizes the time and energy signal of a photo-sensor channel by using two time-to-digital converters (TDCs) and a charge integrator. The digitized signals were stacked as event data, which have information on time tag, channel identifier, and measured energy, through a module referred to as the global controller (FEB/D_V2 board, PETsys Electronics Inc., Oeiras, Portugal). Event data were transmitted to a computer system using a data acquisition board equipped with a field-programmable gate array (FPGA Kintex-7, Xilinx Inc., San Jose, CA, USA). The components of the experiments listed above are shown in Figure 2 (left) and these components were assembled and installed in light-tight structures for the experiment, as shown in Figure 2 (right).

The experiment was performed at a controlled temperature with the air temperature set to 18 °C. Temperature is one of the important SiPM characterization factors because it has significant temperature dependency. Accordingly, SiPM and ASIC temperatures were stable at about 20 and 25 °C, respectively. The breakdown voltage and overvoltage of SiPM were set to 27 and 4 V. ASIC calibration under these settings was performed to reject unnecessary noise signals.

### 2.2. Monte Carlo Simulation

A Monte Carlo simulation was conducted with Monte Carlo N-Particle Transport Code 6, version 1.0 (MCNP 6.1). MCNP6.1 is widely used for modeling the spectroscopy of radiation detectors because this code enables each modeled detector to obtain the pulse height from the emitted particles. To calculate the variations of the transferred energy according to the scintillator interaction with gamma rays of ^176^Lu, an identical detector module to that in the experiment was modeled in the simulation. Moreover, cascade gamma rays, as an evenly distributed radiation source, were inserted in the scintillator. In the case of the beta particles from ^176^Lu, we assumed that their energy was locally deposited in the scintillator.

The energy resolution of the full-energy peak in the pulse-height spectrum measured with a scintillation detector based on SiPM is normally determined by a combination of the physical uncertainties. However, the Monte Carlo simulation used in this study cannot directly take into account the uncertainty factors. Hence, a special treatment, referred to as Gaussian energy broadening (GEB) treatment as a Gaussian kernel, was used to better simulate the detector system [22]. The treatment broadens the energy peaks by sampling from the following Gaussian function:

(1) $$f\left(E\right)=Ce^{-{\left(\left(E-E_{0}\right)/A\right)}^{2}}$$

where *E* is the broadened energy; *E*_0_ is the unbroadened energy of the peaks; *C* is a normalization constant; and *A* is the Gaussian width.

The Gaussian width is related to the full width at half maximum (*FWHM*) by

(2) $$A=\frac{FWHM}{2\sqrt{\ln2}}$$

The *FHWM* is defined as the energy-dependent non-linear response in this code and the expressions of the *FHWM* are shown below. The parameters *a*, *b*, and *c* were derived from experiments with the standard gamma sources (^137^Cs, ^22^Na, and ^133^Ba).

(3) $$FWHM=a+b\sqrt{E+cE^{2}}$$

In this study, the least-square fitting with a gradient descent method was used for acquiring the desired parameters by defining the loss function with *a*, *b*, and *c*. Parameters used for this study are 0.006555456 for “*a*”, 0.060221652 for “*b*”, and 1.130257109 for “*c*”.

### 2.3. Spectra Processing

#### 2.3.1. Definition of Source Pathway and Energy Transfer Cases

The base spectra that constitute the IRL spectrum were derived based on source pathways and interaction cases, as reported previously [16]. Although in some ways the approach we used in the present work is similar to previously reported methods, we subdivided the transferred energy when gamma rays were absorbed in the crystal to address all the relevant physical processes. In the case of the internal conversion in the source pathways, a process in which an excited nucleus transfers its excitation energy directly to an atomic electron, we assumed that its energy is also locally deposited in the scintillator. When gamma rays are emitted as a result of the isomeric transition, each gamma ray interacts independently with the material and transmits its energy to the scintillator. Thus, source pathways, by combinations of the isomer transition pathway, are defined as shown in Table 1 and the resulting combinations of energy transfer cases are defined as shown in Table 2. The amount of energy transfer of each gamma in each case was induced by the Monte Carlo simulations.

#### 2.3.2. Data Processing with Discrete Convolution

We represented the amount of energy transfer as the energy spectrum and conducted data processing according to the combinations to obtain the total amount of energy from the simultaneously independent interactions. Internal conversion in the source pathways contributed to the energy spectrum as part of the total energy peak.

To process the spectra according to the cases, we conducted convolution between energy spectra. All cases have a contribution of the beta particle; thus, the probabilities of the beta spectrum were exploited to fulfill the role of the finite impulse response filter (FIR, *h*[*n*]) in the calculation. Therefore, the probabilities of the gamma spectra were used for input (*x*[*n*]) in order to derive an output (*y*[*n*]). In this process, FIR has values only at finite intervals and the lower bound of energy bins was considered as the representative energy of the energy bin.

(4) $$y\left[n\right]=h\left[n\right]*x\left[n\right]=\overset{n}{\underset{k=1}{\sum }}h\left[n-k\right]\cdot x\left[k\right]$$

*S_β_* denotes the beta particle spectrum; $S_{1}$, $S_{2}$, and $S_{3}$ denote each gamma ray spectra from the Monte Carlo simulation; and *S_e_* denotes the measured spectrum. The energy scale of all the spectra was discretized into an identical number of channels (*n*) and $S_{\beta }\left(k,p\right)$, $S_{1,2,3}\left(k,p\right)$, and $S_{e}\left(k,p\right)$ are the normalized probability of the *k*th channel. To implement the combination of each spectrum, convolution was conducted according to the following:

(5) $$\begin{array}{c} C_{1}\left(k,\;p\right)=S_{\beta }\left(k,p\right)\\ C_{2}\left(k,p\right)=S_{\beta }\left(k,p\right)*S_{1}\left(k,p\right)\\ \vdots \\ C_{7}\left(k,p\right)=S_{\beta }\left(k,p\right)*(S_{2}\left(k,p\right)*S_{3}\left(k,p\right))\\ C_{8}\left(k,p\right)=S_{\beta }\left(k,p\right)*(S_{1}\left(k,p\right)*S_{2}\left(k,p\right)*S_{3}\left(k,p\right)) \end{array}$$

The Gaussian broadened spectrum used here is defined as follows:

(6) $$C_{i}^{\prime }\left(k,p\right)=C_{i}\left(k,p\right)*f\left(k\right),\;i=1,\;2,\;\cdots ,8.$$

An IRL spectrum can then be produced by considering the weights of each case.

(7) $$S_{g}\left(k,p\right)=\overset{8}{\underset{i=1}{\sum }}w_{i}C_{i}^{\prime }\left(k,p\right)$$

It can be seen that particular weight coefficients $w_{i}$ in Equation (7) are needed to generate a proper spectrum. Thus, we calculated the weight coefficients through the measured spectrum. In this paper, a non-linear least-squares optimization method was used to estimate the weight coefficients using the following objective functions:

(8) $$f\left(w_{i}\right)=\sqrt{\frac{r\left(w_{i}\right)}{n}},\;\;\;\;\;\;r\left(w_{i}\right)=\overset{n}{\underset{p=1}{\sum }}{\left\{S_{e}\left(k,p\right)-S_{g}^{\prime }\left(k,p\right)\right\}}^{2}$$

where $S_{g}^{\prime }\left(k,p\right)$ is the re-organizing spectrum calculated with $w_{i}$, which indicates it makes a contribution to the spectrum. The proper values of $w_{i}$ should minimize the difference between $S_{e}\left(k,p\right)$ and $S_{g}^{\prime }\left(k,p\right)$. The optimized group of $w_{i}$, $G_{opt}\left(w_{i}\right)$ can be calculated by solving the following optimization problem:

(9) $$G_{opt}\left(w_{i}\right)=arg\underset{w_{i}}{\min}f\left(w_{i}\right)$$

There are many methods to solve a non-linear optimization problem. In this study, the genetic algorithm (GA) was employed to solve the problem since it is not easy to define the existence of the local optimal solution. The algorithm, as an optimization solver based on a stochastic process derived from an evolutionary model, with operations such as selection, crossover, and mutation, could derive a global optimal solution, thus escaping the local optimal solution by using these operations [23,24].

## 3. Results and Discussion

### 3.1. Isomer Transition Spectra

#### 3.1.1. Calculation of the Total Photo-Peak Ratio

The interaction of IRL with the LYSO scintillator was calculated using a Monte Carlo simulation. In addition, the results of the simulation were modified based on the following equation considering the pathway yield of the isomer transition, which was assumed in advance.

(10) $$T=\frac{\left(Y_{\gamma }\cdot P_{\gamma }\cdot E_{\gamma }\right)+\left(Y_{IC}\cdot P_{IC}\cdot E_{IC}\right)}{(Y_{\gamma }\cdot P_{\gamma })+\left(Y_{IC}\cdot P_{IC}\right)}$$

In the equation, the total peak ratio (*T*) of the isomer transition was induced by the yield (*Y*), interaction probability (*P*), and full-energy peak (or photo-peak) efficiency (*E*). The interaction probability represents the interaction probability of the radiation emitted from the isomer transition with the scintillator. In the case of the gamma ray emission, the interaction probability varied because the energy was different according to the isomer transition phase. For the internal conversion, however, all probabilities were expressed as 1 because the electrons from the internal conversion were locally deposited. The photo-peak efficiency represents the probability of total absorption when the radiation interacted with the scintillator. The total peak ratio considering all paths of each isomer transition based on these calculations is shown in Table 3.

Although this study used a pixelated small crystal (3 × 3 × 20 mm^3^), the relatively bulky crystals (10 × 10 × 10 mm^3^ and 30 × 30 × 30 mm^3^) used in previous studies and the much smaller crystal (3 × 3 × 3 mm^3^) were also simulated to calculate and compare the total peak ratio of each isomer transition. As shown in Table 4, it can be seen that as the size of the crystal increases, the ratio of the peak ratio increases. This suggests that if the crystal is sufficiently bulky, there is no significant difference in the IRL spectrum even if all the interactions are considered as the full-energy peak. In addition, this study adopted a ^176^Lu decay scheme with relatively low gamma emission yields, as presented in existing papers. Accordingly, if a value other than the decay scheme used in this manuscript is used, the photo-peak ratio may decrease.

#### 3.1.2. Post-Processing Isomer Transition Spectra

**Figure 3:**
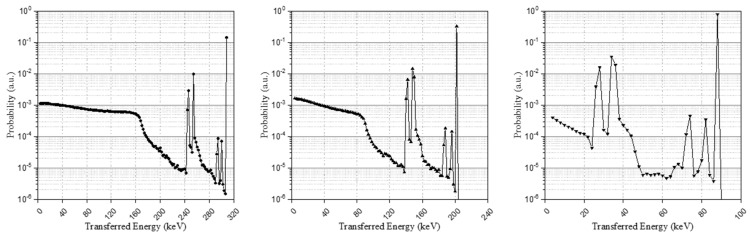
Isomer transition I, II, and III spectra in the LYSO crystal with a size of 3 × 3 × 20 mm^3^.

The post-processing results of the simulation, considering all source pathways, are shown as an energy spectrum in Figure 3 and each energy spectrum represents each isomer transition result. Broadening had not yet been applied to the energy spectra through the pre-defined Gaussian kernel and the base spectra will be configured through data processing in the next section.

### 3.2. Base Spectra

The isomer transition energy spectra were processed by the combination of cases to configure the base spectra that form the IRL spectrum. In this study, the Gaussian kernel used for spectrum broadening was defined using energy resolutions of radiation sources as empirical data in the indirect radiation measurement using a scintillator and SiPM [25,26]. In these measurement systems, the energy resolution (ΔE/E) can be expressed as follows:

(11) $${\left(\Delta E/E\right)}^{2}={\left(\delta_{sc}\right)}^{2}+{\left(\delta_{p}\right)}^{2}+{\left(\delta_{st}\right)}^{2}+{\left(\delta_{n}\right)}^{2}$$

where $\delta_{sc}$ is the intrinsic resolution of the scintillator, $\delta_{p}$ is the transfer resolution, $\delta_{st}$ is the statistical contribution of a silicon photomultiplier, and $\delta_{n}$ is the noise contribution. The interaction by each isomer transition contributes to the signal simultaneously but each interaction is mutually independent. In other words, if broadening is performed on the energy merged after convolution, it is possible that the system performance may not be accurately reflected because the energy resolution is also an energy-dependent non-linear response [27]. Thus, we tried to confirm these concerns through a comparison of the two methods. One comparison is to apply the Gaussian broadening after the convolution, as described in Section 2.3.2 (GBAC), and another is to apply the Gaussian broadening before the convolution, as described below the following equation (CAGB).

(12) $$S_{i}^{\prime }\left(k,p\right)=S_{i}\left(k,p\right)*f\left(k\right),\;i=\beta ,\;1,\;2,\;3$$

**Figure 4:**
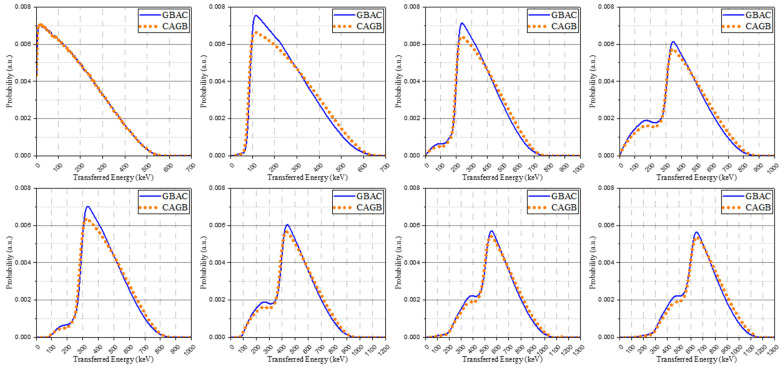
The base spectra obtained by two methods: Gaussian broadening after convolution (GBAC, blue solid line) and convolution after Gaussian broadening (CAGB, orange dot line).

The isomer transition spectra modified through Gaussian broadening replaced the primary spectra and created the base spectra in the same way. The base spectra obtained by these methods were normalized to have identical areas. There was no significant difference in most spectra according to the order of convolution and Gaussian broadening. Through this process, however, we confirm that when simultaneously contributing to a signal through independent interactions, there could be a difference in the low-energy area in which the performance of the system is relatively degraded. The spectra from two methods are described in Figure 4 and GBAC-based spectra were used in the discussion of the remaining results.

**Figure 5:**
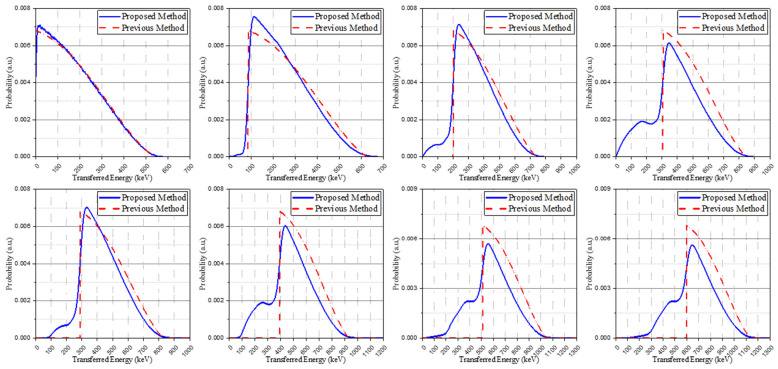
Base spectra of the proposed method (blue solid line) and base spectra of the previous method (red dot line) for configuring the IRL spectrum.

Figure 5 shows the base spectra derived from the proposed method (GBAC) in this manuscript and the base spectra used in previous studies [15]. The proposed method considered Compton-scattering using the isomer transition spectra (blue solid line), whereas the previous method does not consider Compton-scattering (red dash line). For the base spectra used in previous studies, no post-processing was carried out.

### 3.3. Validation of the Study

#### 3.3.1. Genetic Algorithm-Based Spectrum Configuring

**Figure 6:**
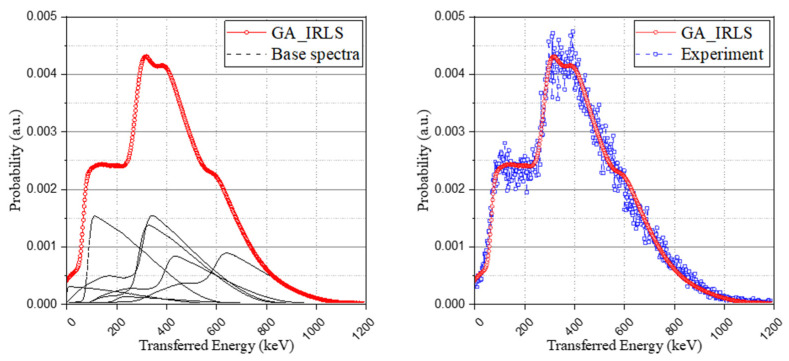
Configuring the genetic algorithm-based IRL spectrum (GA_IRLS) with weighted base spectra (**left**) and the validation of the study with experimental IRL spectrum (**right**).

The experimental spectrum and base spectra were normalized to have identical areas and to be used as the input of the objective function. The objective function for optimizing the spectrum was solved using a genetic algorithm. The results derived from the calculation were the weight coefficients that minimize the root-mean square error (RMSE) of the simulated and experimental spectrum within specific conditions. Thereby, based on Equation (7), the IRL spectrum of the simulation was generated by the sum of the multiplication of the optimized weight coefficients and the corresponding base spectrum. As can be seen in Figure 6, the simulation spectrum was generated in good agreement with the experimental spectrum.

#### 3.3.2. Interaction Probability-Based Spectrum Configuring

**Figure 7:**
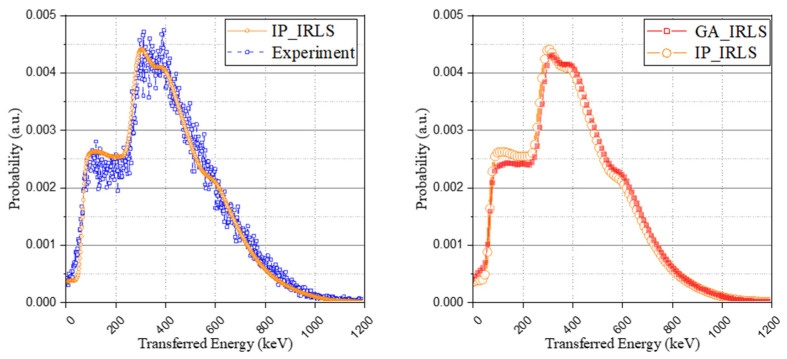
Interaction probability-based IRL spectrum (IP_IRLS) with experimental IRL spectrum (**left**) and comparison of the two approaches (**right**).

In addition to the approach of the optimization problem using the genetic algorithm, another method using relative intensity calculation with a Monte Carlo simulation to derive the possibility of the occurrence of each case was used to generate the IRL spectrum. The interaction probability and the escape probability of each radiation according to the isomer transition phase were taken into account (the results were set out in Table 5), and the method also used the same base spectrum as used in the optimization method and normalized it to have an identical area. As shown in Figure 7, although the retrospective method using the genetic algorithm provides a more detailed spectrum than the relative intensity calculation approach, both methods have good agreement with the experimental spectrum.

The probability of all cases may not be ideal during the experiment, unlike a simulation, and the following uncertainties can produce differences between the two approaches. Accordingly, this suggests that when the insufficient IRL spectrum is used for various purposes, the probability of the occurrence of each case can be extracted more precisely with optimization methods such as the genetic algorithm.

#### 3.3.3. Comparison of the Proposed Method without the Compton Model and GATE Simulation Package

**Figure 8:**
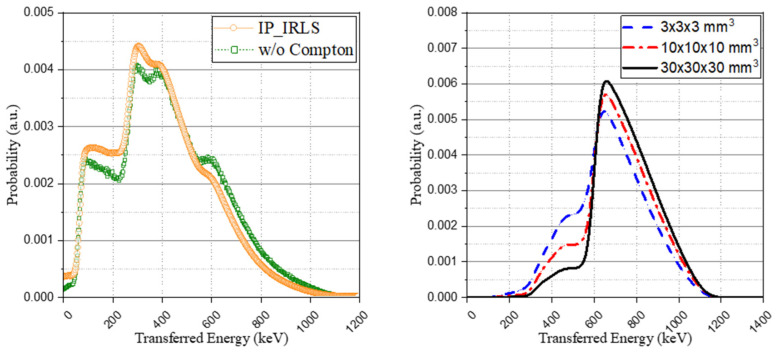
Comparison of the method without the Compton model (previous studies) (**left**) and base spectra of the eighth case depending on crystal structures (**right**).

A comparison of the method without the Compton model, as proposed from previous studies, was performed. For this, the based spectra of Figure 5 and the weight coefficient derived from the interaction probability calculation were used to generate the IRL spectrum. Although a general agreement was achieved with both spectra, showing similar trends, as depicted in Figure 8 (left), there was a difference in the peaks of the spectrum. This is because, as shown in Figure 8 (right), the previous method that considers all the interactions from the isomer transition, including gamma ray emission or internal conversion as a total energy peak, overestimates the peak intensity when the scintillator structure becomes smaller.

**Figure 9:**
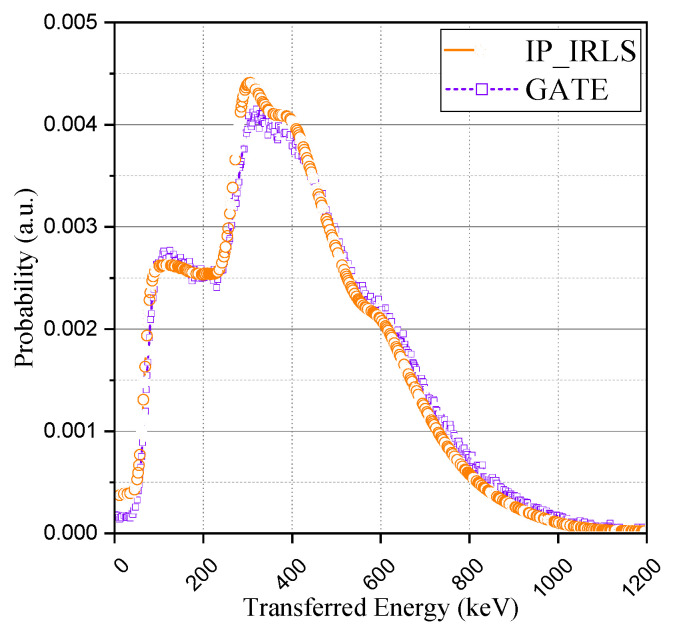
Comparison with GATE simulation (^176^Lu ion source).

Finally, the comparison was made using the ^176^Lu ion source of the GATE (Geant4 Application for Tomographic Emission) simulation package. Considering that the simulation package also does not include this intrinsic radioactivity by default in its Lutetium-based scintillator model [19], we evenly distributed the ion source in the crystal volume. The result from the GATE simulation was also discretized into an identical number of channels and normalized. As can be seen in Figure 9, the acquired spectrum with GATE was in good agreement with the proposed method and experimental spectrum. The results of the GATE simulation and the proposed method have something in common; the IRL spectrum can be acquired in consideration of Compton-scattering. It is a great advantage to be able to insert the intrinsic radioactivity of lutetium into the Monte Carlo simulation because it could be usefully utilized for various studies based on its radioactivity. This study derived the base spectra of the IRL spectrum considering the effect of Compton-scattering, which was a limitation of previous studies, instead of the IRL spectrum obtainable from the results of the experiment. We believe that these results will enable a more delicate approach in applications such as energy calibration and system performance evaluation.

## 4. Conclusions

A method for deriving the spectrum from intrinsic radiations of ^176^Lu considering the various physical process has been proposed. Base spectra configuring the IRL spectrum were generated by combining three isomer transition spectra and the spectrum of the β-particle. Using two approaches, namely the optimization problem and relative intensity calculation, contributions of each base spectrum were calculated. From the results, we confirm that both approaches using the proposed methods provide good agreement with the experiment.

A significant contribution of this study is the provision of a new approach to process the energy spectra induced by simultaneously detecting the multiple signals of mutually independent radiation interactions in the system. Formerly, a simple summation of the emitted energy from the isomeric transition was used to generate the spectrum formed by the interactions of the intrinsic radiation of lutetium. In this study, however, all the physical processes in the scintillator crystal were reflected using spectrum convolution. Furthermore, we confirmed that the structure of the scintillator not only causes differences in the contribution of the cases but also produces differences in the base spectra that make up each case.

As a result, base spectra, in which Compton-scattering was considered, were derived and the validation of the results was confirmed by the experiments and simulation package. We believe that this achievement could provide more detailed information for cases when the intrinsic radiation of ^176^Lu is utilized, such as in energy calibration and system performance evaluations without external radiation sources. Furthermore, this study suggests that necessary data can be extracted and utilized by solving optimization problems using appropriate constraints for the non-ideal IRL spectrum even when the data is insufficient.

## Figures and Tables

**Table 1:** The source pathway by combinations of the isomer transition pathway.

Source Pathway	Cases of Gamma Ray Emission (γ) or Internal Conversion (IC) by Isomer Transition (IT)		
	307 keV (IT_I_)	202 keV (IT_II_)	88 keV (IT_III_)
1	γ_1_	γ_2_	γ_3_
2	γ_1_	γ_2_	IC_3_
3	γ_1_	IC_2_	γ_3_
4	γ_1_	IC_2_	IC_3_
5	IC_1_	γ_2_	γ_3_
6	IC_1_	γ_2_	IC_3_
7	IC_1_	IC_2_	γ_3_
8	IC_1_	IC_2_	IC_3_

**Table 2:** The cases of energy deposition by the state of ionizing radiation from the isomer transition.

Energy Deposition Cases	Beta Particle	State of Ionizing Radiation		
		Isomer Transition I	Isomer Transition II	Isomer Transition III
1	Interaction	Interaction	Interaction	Escape
2	Interaction	Interaction	Interaction	Interaction
3	Interaction	Interaction	Escape	Escape
4	Interaction	Interaction	Escape	Interaction
5	Interaction	Escape	Interaction	Escape
6	Interaction	Escape	Interaction	Interaction
7	Interaction	Escape	Escape	Escape
8	Interaction	Escape	Escape	Interaction

**Table 3:** Total photo-peak ratio by isomer transitions.

Isomer Transition Pathway	Isomer Transition I		Isomer Transition II		Isomer Transition III	
	γ_1_	IC_1_	γ_2_	IC_2_	γ_3_	IC_3_
Yield (*Y*)	0.931	0.069	0.780	0.220	0.146	0.854
Interaction probability (*P*)	0.224	1.000	0.399	1.000	0.863	1.000
Photo-peak efficiency (*E*)	0.641	1.000	0.818	1.000	0.909	1.000
Total photo-peak ratio (*T*)	0.730		0.893		0.988	

**Table 4:** Dependency of the total photo-peak ratio according to the crystal structures.

Crystal Size	Isomer Transition I	Isomer Transition II	Isomer Transition III
3 × 3 × 3 mm^3^	0.717	0.880	0.985
10 × 10 × 10 mm^3^	0.809	0.941	0.995
30 × 30 × 30 mm^3^	0.914	0.978	0.998

**Table 5:** Relative intensity calculation by energy deposition case for configuring the spectrum.

	**Isomer Transition I**	**Isomer Transition II**	**Isomer Transition III**
**Interaction**	0.2775	0.5310	0.9800
**Escape**	0.7225	0.4688	0.020
**Energy deposition cases**		**Probability**	
1		0.00677	
2		0.33182	
3		0.00767	
4		0.00260	
5		0.37632	
6		0.12745	
7		0.00294	
8		0.14443	
**Sum**		1.00000	

## Data Availability

Not applicable.
